# Supplementary material for: Exploring the Utility of Cardiovascular Magnetic Resonance Radiomic Feature Extraction for Evaluation of Cardiac Sarcoidosis
Source: Diagnostics (Basel). 2023 May 26;13(11):1865. doi: 10.3390/diagnostics13111865 (PMC10252949; doi:10.3390/diagnostics13111865)
Supplement: Supplementary file 1 [file diagnostics-13-01865-s001.zip › Supplementary Materials File S2 Tables S1 and S2.pdf]

Supplementary Material

Table S1. Area under the curve (AUC) and accuracy of the five best-performing radiomic features based on AUC values before and after correlated features removed. GLCM: Gray Level Co-occurrence Matrix.

	Feature	Accuracy	AUC	β	p-value
Correlation retained	Firstorder_Mean Absolute Deviation	0.70	0.73	0.139	1.25E-08
	Firstorder_ Interquartile Range	0.71	0.72	0.018	1.41E-05
	Firstorder_Robust Mean Absolute Deviation	0.72	0.72	-	-
	GLCM_Contrast	0.70	0.71	-0.001	1.97E-13
	Firstorder_Range	0.71	0.70	-0.185	1.24E-02
Correlation removed	Firstorder_Mean Absolute Deviation	0.70	0.73	0.139	1.25E-08
	Firstorder_ Interquartile Range	0.71	0.72	0.018	1.41E-05
	GLCM_Contrast	0.70	0.71	-0.001	1.97E-13
	Firstorder_Range	0.71	0.70	-0.185	1.24E-02
	GLCM_Joint Entropy	0.72	0.70	-0.389	1.85E-02

Table S2. Area under the curve (AUC) and accuracy of the machine learning classifiers for the different signatures.

Machine Learning Classifier	Signature A		Signature B	
	Accuracy	AUC	Accuracy	AUC
Random Forest	0.61	0.64	0.59	0.76
Logistic Regression	0.67	0.64	0.62	0.68
Support Vector Machine	0.67	0.77	0.54	0.65
Decision Tree	0.59	0.59	0.69	0.69
Gaussian Process Classifier	0.62	0.50	0.52	0.70
Stochastic Gradient Descent	0.53	0.55	0.58	0.61
Perceptron Classifier	0.49	0.42	0.61	0.63
Passive Aggressive Classifier	0.51	0.46	0.62	0.70
Neural Network Classifier	0.48	0.46	0.54	0.62
K-neighbors Classifier	0.72	0.73	0.56	0.64
